# Lignocellulose-Chitosan-Multiwalled Carbon Nanotube Composites with Improved Mechanical Strength, Dimensional Stability and Fire Retardancy

**DOI:** 10.3390/polym10030341

**Published:** 2018-03-20

**Authors:** Zhe Wang, Yutao Yan, Xiaoping Shen, Temeng Qian, Junjie Wang, Qingfeng Sun, Chunde Jin

**Affiliations:** 1 School of Engineering, Zhejiang A&F University, Hangzhou 311300, China; donjade@163.com (Z.W.); yytao1988@163.com (Y.Y.); sxp1031@hotmail.com (X.S.); teemeeng@163.com (T.Q.); jjwang7475@163.com (J.W.); 2 Key Laboratory of Wood Science and Technology, Hangzhou 311300, China

**Keywords:** lignocellulose composites, chitosan, multiwalled carbon nanotube, mechanical strength, dimensional stability, fire retardancy

## Abstract

A novel composite composed of lignocellulose (LC), glutaraldehyde crosslinked chitosan (GC) and multiwalled carbon nanotube (MWCNT) was fabricated by the hot-pressing process. The effect of the additional GC and MWCNT on the mechanical strength, dimensional stability and fire retardancy of lignocellulose composites was investigated. The results showed that LC/GC/MWCNT composite exhibited the maximum modulus of rupture (MOR) of 35.3 MPa, modulus of elasticity (MOE) of 2789.1 MPa and internal bonding (IB) strength of 1.2 MPa. Meanwhile, the LC/GC/MWCNT composite displayed improved dimensional stability with a thickness swelling (TS) value of 22.4%. Besides, the LC/GC/MWCNT composite exhibited improved fire retardancy with a limiting oxygen index of 29.0%. The peak heat release rate, the total heat release, the total smoke production and the maximum smoke production ratio of LC/GC/MWCNT composite decreased by 15.9%, 10.7%, 45.5% and 20.7% compared with those of LC composite, respectively. Therefore, the LC/GC/MWCNT composite may be a promising candidate for green wood based composites.

## 1. Introduction

In recent years, the excessive deforestation of natural forests resulted in the diminishing amount of wood resources [1]. The fabrication of lignocellulose composites based on wood waste, crotches and processing residues is an effective way to saving wood resources. Lignocellulose composites have been widely used in our daily life, such as furniture and interior decoration materials [2]. In general, the use of adhesives determines the mechanical strength of lignocellulose composites during the fabrication process. Among them, the urea formaldehyde and phenol formaldehyde adhesives are the most commonly used. However, it is a serious issue for human health and environment due to the release of free formaldehyde during the fabrication and use of lignocellulose composites. Meanwhile, the production of formaldehyde relies on the non-renewable and diminishing petroleum resource. Thus, there is an insistent demand to develop a green fabrication process of lignocellulose composites via the use of renewable resources.

As renewable resources, bio-based materials have been used for the fabrication of formaldehyde-free adhesives [3], such as lignin and its derivatives [4,5,6], cellulose nanofiber [7,8], plant protein [9,10,11,12], starch [13,14], and so on. However, the above bio-based materials have not been extensively applied due to their complex fabrication processes, poor mechanical properties and dimensional stability. Therefore, there is an urgent need to develop a facile process to fabricate the formaldehyde-free lignocellulose composites with a high mechanical strength and dimensional stability.

Chitosan has attracted an extensive attention due to the nature of renewability and biodegradability [15,16]. Furthermore, chitosan has a rich source because it exists widely in the shell of shrimp and crab [17,18]. A large number of free amino and hydroxyl groups exist in chitosan, which can achieve bonding strengths between materials [18,19]. Recently, some studies have indicated that chitosan may be a promising candidate for the adhesives of wood based materials [20,21,22]. However, the linear nature of chitosan could lead to the deformation of materials. To overcome this structural limitation, the three-dimensional polymer networks of chitosan have been created by the chemical cross-linking with glutaraldehyde [23,24,25,26]. 

In order to ensure the safety use of lignocellulose composites, their flammability needs to be mentioned. Various strategies have been developed to improve the fire retardancy of lignocellulose composites, such as coating or soaking with fire retardants [27,28,29]. With the increase of the usage time, fire retardant coatings are easily damaged resulting in the decrease of fire retardancy. Furthermore, the leakage of hazardous fire retardants is also a serious issue for human health and environment [30,31]. Thus, there is an urgent demand to develop an environmentally friendly method to improve the fire retardancy of lignocellulose composites. Some studies have indicated that carbon nanotube is an efficient and nontoxic fire retardant [32,33,34]. In addition, the tensile strengths and Young’s modulus of the carbon nanotube are obviously higher than those of stainless steel [35,36]. The addition of carbon nanotube is beneficial to improving mechanical strengths of materials [37,38].

In this study, we fabricate a novel lignocellulose/crosslinking chitosan/multiwalled carbon nanotube composite by the conventional hot pressing process. On the one hand, a green and formaldehyde-free composite with excellent bending and internal bonding strengths was prepared using renewable and biodegradable chitosan as the binder. On the other hand, the addition of multiwalled carbon nanotube further improved the mechanical strengths and endowed the composite with flame retardancy and smoke suppression. This study is believed to introduce a new route to fabricate multifunctional and formaldehyde-free lignocellulose composites. It is also believed that the applications of lignocellulose composites with high mechanical strength, flame retardancy and smoke suppression will be expanded in real life.

## 2. Materials and Methods

### 2.1. Materials

Lignocellulose fibers (20–100 mesh), consisting of a blend of softwood and hardwood fibers from different species, were provided by Great World Group (Ningbo, China). Chitosan (CS, powder, more than 120 mesh, deacetylation degree > 95%, viscosity: 100–200 mPa·s) was purchased from Macklin Biochemical Co., Ltd. (Shanghai, China). Multiwalled carbon nanotube (MWCNT, Purity: 90%, Product No. XFIM4) was purchased from Xianfeng Nanotech Co., Ltd. (Nanjing, China). Acetic acid was supplied by Lingfeng Chemical Reagent Co., Ltd. (Shanghai, China). Glutaraldehyde (25 wt %) was provided by Sinopharm Chemical Reagent Co., Ltd. (Shanghai, China). All chemicals were used as received without any further purification. 

### 2.2. Fabrication of Crosslinked Chitosan/Multiwalled Carbon Nanotube Hydrogel

Crosslinked chitosan was prepared according to the previous studies with minor modifications [25,26]. Firstly, CS was solubilized in an acetic acid solution of 1.5% (*w*/*v*) to obtain a 2% (*w*/*v*) CS mixture at room temperature for 1 h under stirring. Secondly, 2% (*w*/*v*) of multiwalled carbon nanotube (MWCNT) was added to the CS mixture. Then, the above mixture was subjected to ultrasound treatment for 30 min. Finally, glutaraldehyde (20 wt %, based on CS) was dropwise injected into the CS/MWCNT mixture under continuous agitation until the formation of the GC/MWCNT hydrogel.

### 2.3. Preparation of Lignocellulose-Chitosan-Multiwalled Carbon Nanotube Composite

**Figure 1:**
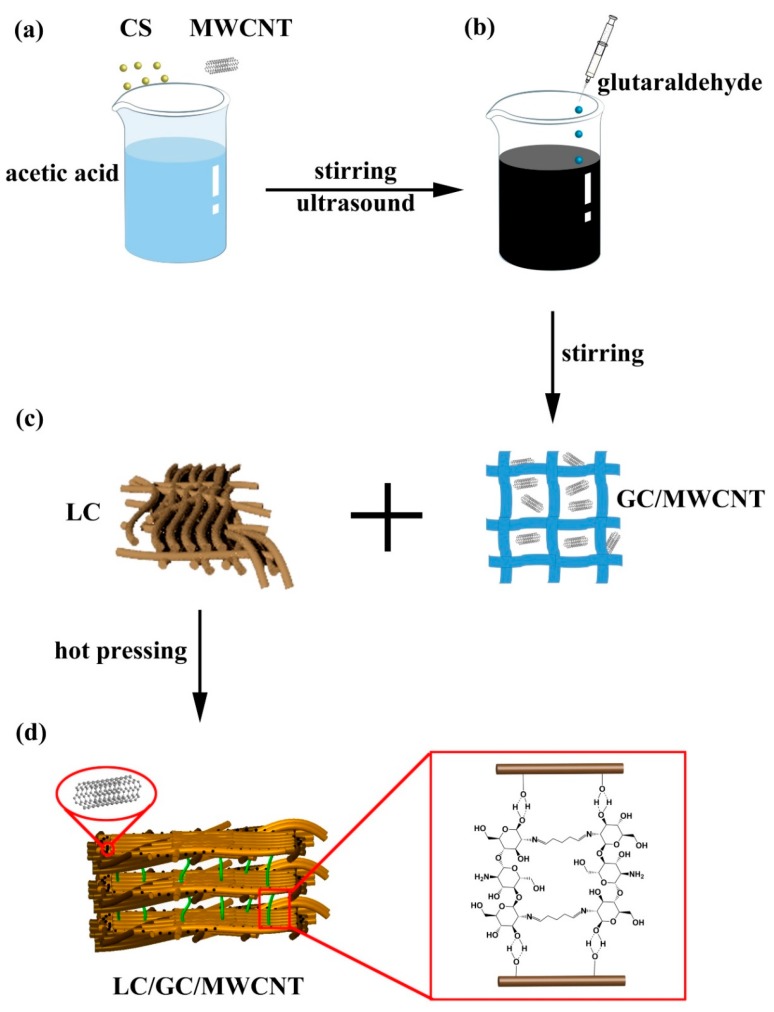
Schematic illustration of the fabrication of LC/GC/MWCNT composite: (**a**) Mixture of CS and MWCNT (**b**) Fabrication of crosslinked chitosan/multiwalled carbon nanotube hydrogel (**c**) Preparation of LC/GC/MWCNT composite (**d**) Preparation mechanism of LC/GC/MWCNT composite.

The fabrication process of LC/GC/MWCNT composite is illustrated in Figure 1. The hot pressing process was used to fabricate lignocellulose composites. Before hot pressing process, the lignocellulose fibers and GC/MWCNT hydrogel were evenly mixed together in a mass ratio of 5.0:100 (CS to lignocellulose fibers). Afterwards, the blended fibers were hot pressed under 180 °C temperature, 4.5 MPa pressure, and 9 min pressing time to form a board with a size of 200 mm × 200 mm × 3 mm. The target density of the lignocellulose composites was 0.83 ± 0.02 g/cm^3^. As a comparison, the same amounts of water, CS and GC were mixed with lignocellulose fibers to fabricate LC composite, LC/CS composite and LC/GC composite by the above same process, respectively.

### 2.4. Physical and Mechanical Properties Test

The bending strengths of lignocellulose composites were measured on a universal mechanical testing machine (Instron 5960, Instron Corporation, Norwood, MA, USA) following GB/T 17657-2013 (Chinese National Standard). In order to investigate the MOR and MOE values of lignocellulose composites with dimensions of 180 mm × 50 mm × 3 mm, the three-point bending measurements were carried out on the samples via a loading speed of 5 mm min^−1^. Impact toughness (IT) test of lignocellulose composites with a dimension of 80 mm× 10 mm × 3 mm was carried out on a pendulum tester (ZBC1000, MST, Eden Prairie, MN, USA) according to GB/T 1843-2008 (Chinese National Standard). Vertical tensile test was performed on the samples with a dimension of 50 mm × 50 mm × 3 mm by a loading speed of 1.0 mm min^−1^ to evaluate the IB values. The measurements of 24 h thickness swelling (TS) were carried out to determine the dimensional stability of lignocellulose composites. 12 samples were repeated for the MOR, MOE and IT tests and 8 samples were repeated for the IB and 24 h TS measurements.

### 2.5. Characterization

Scanning electron microscopy (SEM, TM3030, Hitachi, Tokyo, Japan) was used for the observation of the surface morphologies of lignocellulose composites at an accelerating voltage of 15.0 kV. Transmission electron microscopy (TEM) images of MWCNT were obtained on Tecnai G20. X-ray diffraction (XRD) measurements were carried out on D8 Advance to identify the changes of crystalline structures using Cu Kα (λ = 1.5418 Å) at a scan rate (2θ) of 4° min^−1^ and the accelerating voltage of 40 kV and the applied current of 30 mA ranging from 10° to 80°. Fourier transform infrared (FTIR) spectroscopy were recorded on a Nicolet 460 spectrometer via a KBr pellet pressing method in the wavenumber range of 400–4000 cm^−1^ at a resolution of 4 cm^−1^. The surface chemical compositions of LC composite and LC/GC/MWCNT composite were investigated using X-ray photoelectron spectroscopy (XPS, Thermo ESCALAB 250XI, Waltham, MA, USA). Thermogravimetric (TG) analysis was performed on a Q500 analyzer (TA Instruments, New Castle, DE, USA) from 20 to 700 °C at a heating rate of 10 °C min^−1^ under a N_2_ atmosphere. The limiting oxygen index tests of 15 repeated lignocellulose composites samples with a dimension of 150 mm × 10 mm × 3 mm were carried out by a JF-5 oxygen index instrument. Fire retardancy of LC/GC/MWCNT composite samples with dimensions of 100 × 100 × 3 mm^3^ was investigated using cone calorimeter (FTT Ltd., Derby, UK) at a 50 kW m^−2^ irradiance. The test was repeated three times for each sample.

## 3. Results and Discussion

### 3.1. Micromorphology of Composites

**Figure 2:**
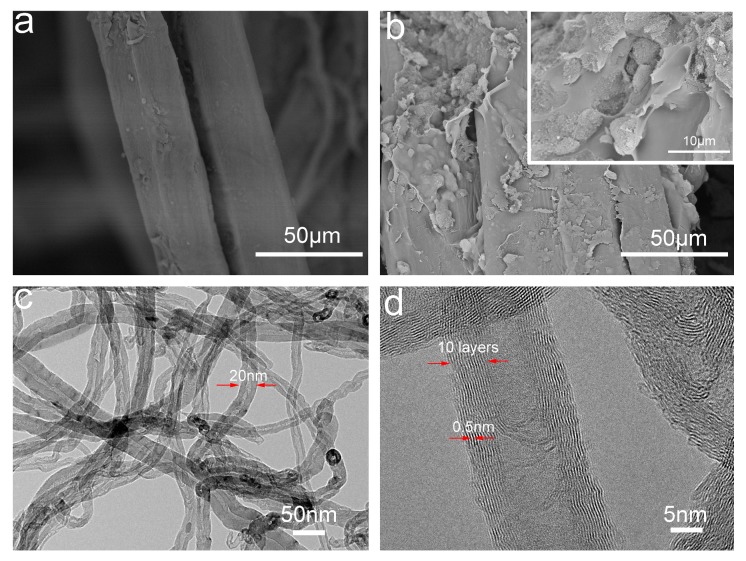
SEM images of LC composite (**a**); LC/GC/MWCNT composite (**b**); TEM images of MWCNT (**c**,**d**).

Figure 2a,b show the fiber surface morphologies of LC composite and LC/GC/MWCNT composite. As shown in Figure 2a, a clear gap between the two fibers was observed, indicating the loose structure of LC composite. This could lead to the poor mechanical properties of LC composite. In addition, the fiber surface of LC composite displayed a typically rough microstructure of lignocellulose cell wall, which was conducive to the enhancement of mechanical interlocking between the GC and lignocellulose fibers [2]. Thus, a better mechanical property may be obtained by the enhanced mechanical interlocking. As shown in Figure 2b, the surface of LC/GC/MWCNT composite exhibited a rougher morphology after the addition of GC/MWCNT hydrogel, indicating that the GC/MWCNT was successfully attached to the lignocellulose fiber surface. Furthermore, the filamentous morphology of MWCNT can be observed on the lignocellulose fibers in Figure 2b insert. Meanwhile, a tighter structure was observed due to the bonding effect of the GC at the interface between the two fibers. As can be seen from the TEM images of MWCNT (Figure 2c,d), the average diameter, wall layers number and average wall thickness of MWCNT was about 20 nm, 10 nm and 0.5 nm, respectively.

### 3.2. X-ray Diffraction Analysis

**Figure 3:**
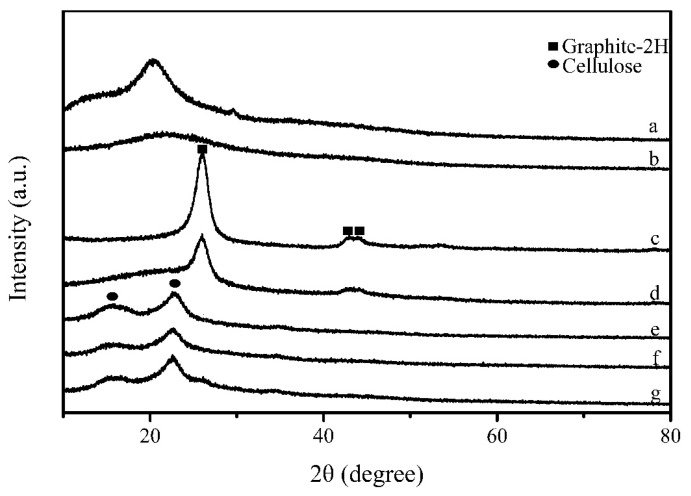
XRD patterns of CS (**a**); GC (**b**); MWCNT (**c**); GC/MWCNT (**d**); LC composite (**e**); LC/GC composite (**f**); LC/GC/MWCNT composite (**g**).

Figure 3 presents the XRD patterns of CS, GC, MWCNT, GC/MWCNT, LC composite, LC/GC composite and LC/GC/MWCNT composite. As shown in Figure 3a, CS exhibited two characteristic peaks at 12.5° and 20.5°. After reacted with glutaraldehyde, the resulting GC samples only displayed a relatively weak characteristic peak at 22.1° (Figure 3b), indicating that the additional glutaraldehyde decreased the orderliness of CS structures [24,26]. Meanwhile, a three-dimensional network structure could be obtained due to the change in the linear nature of CS [26]. Figure 3c exhibited three diffraction peaks at 26.0°, 42.2° and 44.3° assigned to the (002), (100) and (101) planes of MWCNT [39,40], which were consistent with graphite-2H (PDF#41-1487). As shown in Figure 3d–f, three diffraction peaks at 15.5°, 22.8° and 34.5° assigned to the crystalline structure of cellulose were observed in LC composite, LC/GC composite and LC/GC/MWCNT composite. However, the LC/GC/MWCNT composite showed an additional diffraction peak at 26.0° corresponding to MWCNT crystalline structures, indicating that MWCNT was attached to the lignocellulose fiber surface.

### 3.3. FTIR Analysis

**Figure 4:**
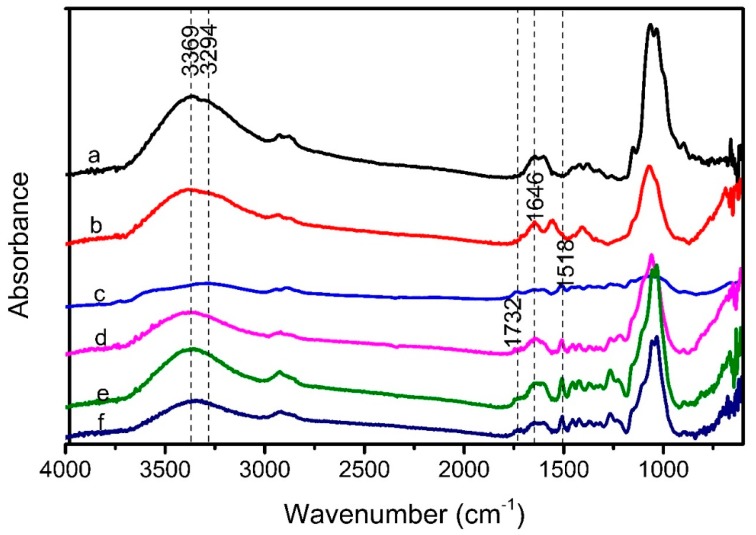
FTIR spectra of CS (**a**); GC (**b**); GC/MWCNT (**c**); LC composite (**d**); LC/GC composite (**e**); LC/GC/MWCNT composite (**f**).

Figure 4a,b show the FTIR spectra of CS before and after the addition of glutaraldehyde. As can be seen from Figure 4a,b, two absorption peaks at 3369 cm^−1^ and 3294 cm^−1^ assigned to –OH stretching vibration and –NH stretching vibration of CS transformed into an absorption peak at 3360 cm^−1^ after the addition of glutaraldehyde, indicating that an interaction between the –NH band of CS and the glutaraldehyde occurred [41]. For the GC sample, an obvious absorption peak was observed at 1646 cm^−1^ attributed to the formation of the C=N bond of the Schiff’s base structure due to the interaction between the –NH band of CS and the C=O groups of glutaraldehyde [25]. This further indicated that the cross-linking reaction between chitosan and glutaraldehyde occurred. In the case of the GC/MWCNT sample (Figure 4c), an absorption peak was observed at 1518 cm^−1^ assigned to the carbon skeleton vibration of carbon nanotubes [42], indicating that the MWCNT was successfully mixed with the GC. 

The FTIR spectra of LC composite, LC/GC composite and LC/GC/MWCNT composite is shown in Figure 4d–f. As shown in Figure 4d–f, similar absorption peaks were observed for LC composite, LC/GC composite and LC/GC/MWCNT composite, which were mainly assigned to the absorption peaks of lignocellulose cell wall. The absorption peak of the –OH stretching vibrations shifted from 3385 cm^−1^ (LC composite) to 3374 cm^−1^ (LC/GC composite) and 3370 cm^−1^ (LC/GC/MWCNT composite), which could result from the hydrogen bond interaction between the GC and the lignocellulose fibers. Some studies also suggested that the formation of bonding strength between lignocellulose fibers and chitosan mainly resulted from the hydrogen bond interaction [26]. Furthermore, for the LC composite, the absorption peak at 1732 cm^−1^ assigned to the C=O stretching vibrations of xylose in lignocellulose could not be observed, which may be due to the thermal degradation of xylose during hot pressing process. However, this absorption peak can be observed in LC/GC composite and LC/GC/MWCNT composite, which could result from the formation of self-polymerized products of glutaraldehyde [26].

### 3.4. XPS Analysis

**Figure 5:**
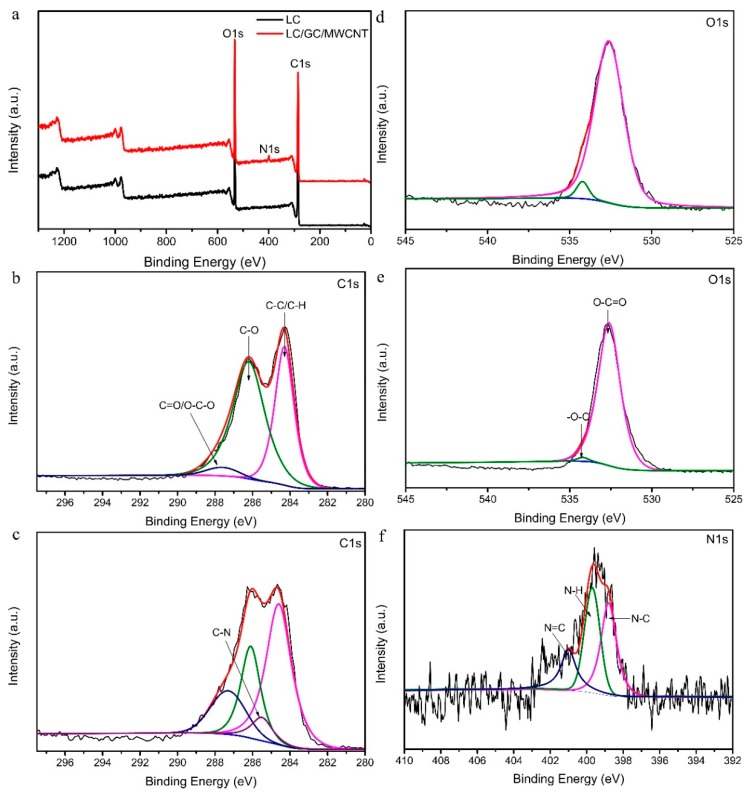
XPS spectra of (**a**) survey spectrum; (**b**) C1s for LC composite; (**c**) C1s for LC/GC/MWCNT composite; (**d**) O1s for LC composite; (**e**) O1s for LC/GC/MWCNT composite; (**f**) N1s for LC/GC/MWCNT composite.

Figure 5 shows the XPS spectra of LC composite and LC/GC/MWCNT composite. As shown in Figure 5a, LC composite and LC/GC/MWCNT composite displayed two main peaks at 285.1 eV and 533.2 eV corresponded to the C1s and O1s, respectively [43]. In the case of LC/GC/MWCNT composite, an additional peak was observed at 399.1 eV corresponded to the N1s [44], indicating that the nitrogen-containing chitosan was attached to the lignocellulose fiber surface. Figure 5b,c show the C1s spectrum of LC composite and LC/GC/MWCNT composite, respectively. As shown in Figure 5b, the C1s spectrum of LC composite exhibited three fitting peaks at 284.6 eV, 286.2 eV and 287.3 eV corresponded to C–C/C–H groups, C–O groups and C=O/O–C–O groups, respectively [45]. Compared with LC composite, LC/GC/MWCNT composite showed an additional peak at 285.5 eV corresponded to C–N groups [46], which further showed that the chitosan was attached to the lignocellulose fiber surface. As shown in Figure 5d,e, the high-resolution O1s spectrum of LC composite and LC/GC/MWCNT composite exhibited two fitting peaks at 532.6 eV and 534.2 eV corresponded to O–C=O groups and C–O– groups, respectively [45]. However, a large decreased peak of C–O– groups was revealed in LC/GC/MWCNT composite, which could result from the addition of MWCNT without C–O– groups. As can be seen from the N1s spectrum of LC/GC/MWCNT composite (Figure 5f), three fitting peaks was obtained at 398.8 eV, 399.3 eV and 400.5 eV corresponded to N–C groups, N–H groups and N=C groups, respectively [47]. The resulting N=C groups of Schiff’s base structure because of the interaction between the –NH band of CS and the C=O groups of glutaraldehyde further confirmed the occurrence of cross-linking reaction of chitosan.

### 3.5. TG Analysis

**Figure 6:**
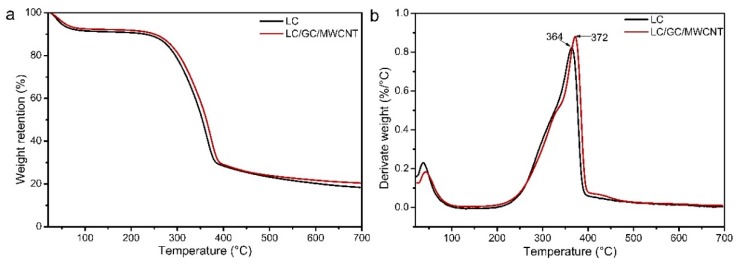
TG-DTG curves of LC composite and LC/GC/MWCNT composite (**a**) TG curves; (**b**) DTG curves.

Figure 6 presents the TG and DTG curves of LC composite and LC/GC/MWCNT composite. As can be seen from Figure 6a, the thermal degradation process of LC composite and LC/GC/MWCNT composite exhibited three stages: (1) the loss of adsorbed water (20 °C to 110 °C); (2) the degradation of polysaccharides in lignocellulose cell wall (110 °C to 400 °C); and (3) aromatization and carbonization of lignin (400 °C to 700 °C) [48,49]. Furthermore, the char residues ratio of LC/GC/MWCNT composite (20.4%) was higher than that of LC composite (17.8%) at 700 °C. The addition of MWCNT may result in the higher char residues ratio of LC/GC/MWCNT composite.

As shown in Figure 6b, the mass loss of LC composite and LC/GC/MWCNT composite mainly occurred in the temperature range from 200 to 400 °C. At this stage, The temperature of endothermic peak increased from 364 °C (LC composite) to 372 °C (LC/GC/MWCNT composite), indicating that an improved thermal stability was obtained for LC/GC/MWCNT composite. 

### 3.6. Mechanical Properties Analysis

**Figure 7:**
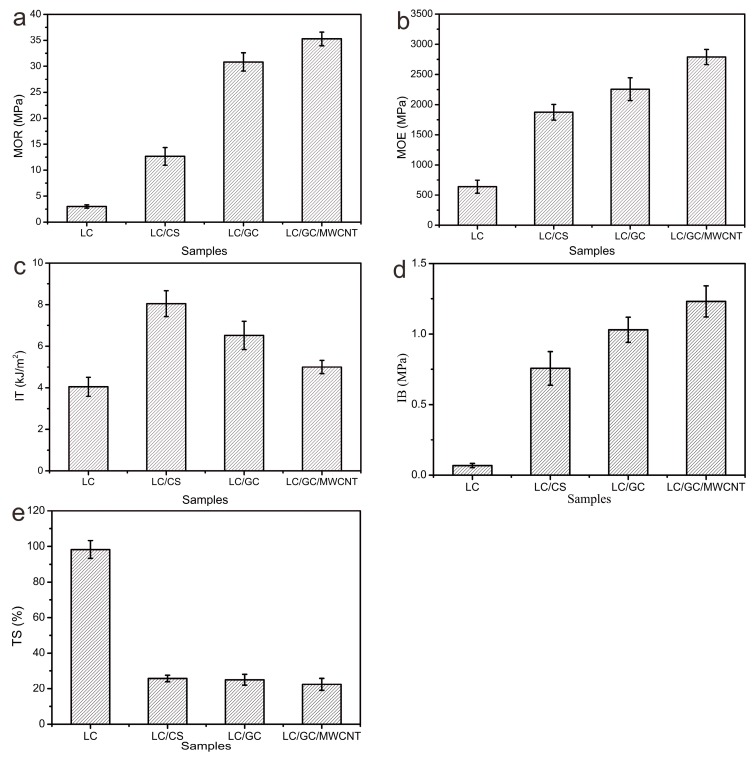
Physical and mechanical properties of LC composite, LC/CS composite, LC/GC composite, LC/GC/MWCNT composite: (**a**) MOR (**b**) MOE (**c**) IT (**d**) IB (**e**) TS values.

Figure 7a–d shows the MOR, MOE, IT and IB of LC composite, LC/CS composite, LC/GC composite and LC/GC/MWCNT composite. As shown in Figure 7a, the MOR value of LC composite was 3.0 MPa. However, the MOR values of LC/CS composite and LC/GC composite increased to 12.7 MPa and 30.8 MPa after the addition of chitosan and crosslinked chitosan, respectively. Interestingly, the additional MWCNT further improved the MOR of lignocellulose composites. The MOR value of LC/GC/MWCNT composite increased to 35.3 MPa and was about three times higher than that of LC/CS composite. As can be seen from Figure 7b, the MOE value of LC composite just reached to 639.3 MPa. The MOE values of LC/CS composite, LC/GC composite and LC/GC/MWCNT composite increased to 1873.9 MPa, 2256.2 MPa and 2789.0 MPa, respectively. The MOR and MOE of LC/GC/MWCNT composite met the minimum requirement for MDF-GP REG of medium density fiberboard (GB/T11718-2009). As shown in Figure 7c, the IT values of LC/CS composite, LC/GC composite and LC/GC/MWCNT composite were higher than those of LC composite. The highest IT value was obtained by LC/CS composite (8.0 kJ m^−2^). However, the IT values decreased after the addition of GC and MWCNT. As shown in Figure 7d, the IB strengths of LC/CS composite, LC/GC composite and LC/GC/MWCNT composite were remarkably higher than those of LC composite. The average IB strengths of LC/GC/MWCNT composite reached up to 1.2 MPa and were two times higher than that of the minimum requirement for MDF-GP REG of medium density fiberboard (GB/T11718-2009). The improvements of LC/GC/MWCNT composite mechanical properties could be attributed to the hydrogen bond interaction and the mechanical interlocking between the GC and the lignocellulose fibers. Furthermore, the addition of MWCNT with high mechanical strengths was beneficial to further improve their mechanical properties.

### 3.7. Dimensional Stability Analysis

The TS values of LC composite, LC/CS composite, LC/GC composite and LC/GC/MWCNT composite are shown in Figure 7e. As shown in Figure 7e, the TS values of LC composite reached up to 98.3% after soaking water for 24 h. However, the TS values of LC/CS composite, LC/GC composite and LC/GC/MWCNT composite decreased to 25.7%, 25.0% and 22.4%, respectively. The LC/GC/MWCNT composite exhibited the lowest TS values in all samples. According to Chinese national standard (GB/T11718-2009, MDF-GP REG), the TS values should be lower than 45% for medium density fiberboard. Therefore, the TS values of LC/CS composite, LC/GC composite and LC/GC/MWCNT composite met this requirement. The Improved dimensional stability of LC/GC/MWCNT composite could be attributed to the bonding effect of chitosan resulting in a decrease in the gaps of lignocellulose composites, which reduced the channels of their water absorption. In addition, the addition of MWCNT as a hydrophobic material could further decrease the water absorption of lignocellulose composites [50,51].

### 3.8. Combustion Test

**Figure 8:**
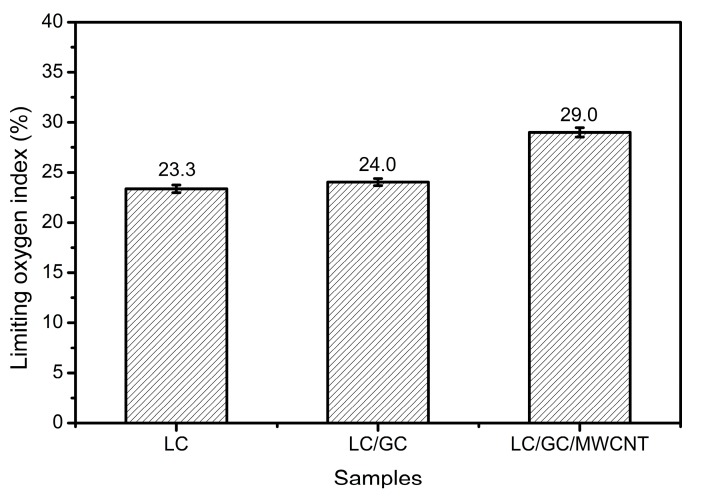
Limiting oxygen index of LC composite, LC/GC composite, LC/GC/MWCNT composite.

The LOI and cone calorimetry tests were carried out to investigate the fire retardancy of LC/GC/MWCNT composite. The higher the LOI values, the better the fire retardancy [48]. As shown in Figure 8, the LOI value increased from 23.3% (LC composite) to 24.0% (LC/GC composite) and to 29.0% (LC/GC/MWCNT composite), indicating that the addition of MWCNT was beneficial to improving the fire retardancy of lignocellulose composites.

**Figure 9:**
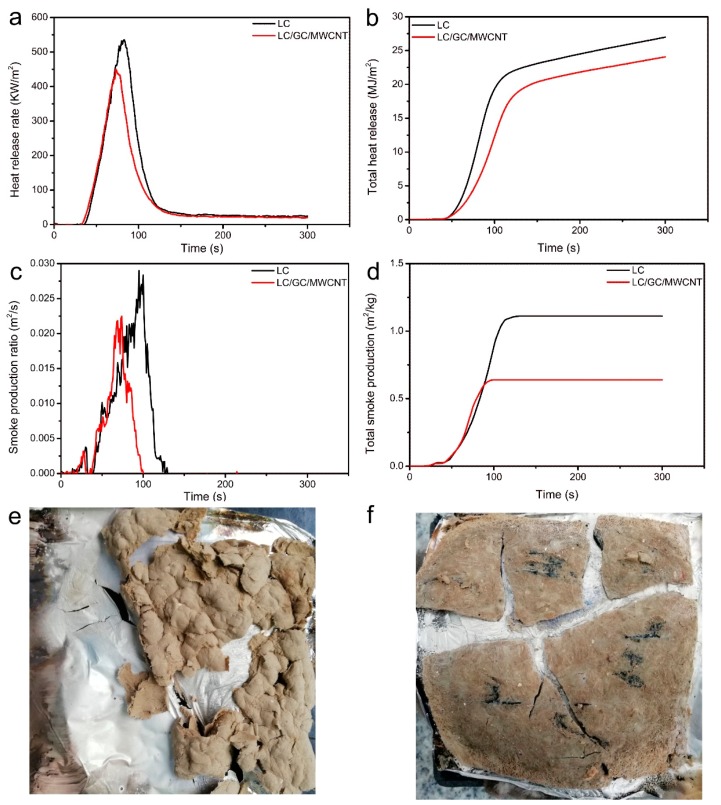
Cone calorimetry curves of LC composite and LC/GC/MWCNT composite: (**a**) heat release rate; (**b**) total heat release; (**c**) smoke production ratio; (**d**) total smoke production; (**e**) residues of LC composite; (**f**) residues of LC/GC/MWCNT composite.

Cone calorimetry measurement was carried out to further evaluate the influence of the additional MWCNT on the fire retardancy of lignocellulose composites. As can be seen from Figure 9a,b and Table 1, the peak heat release rate (PHRR) and total heat release (THR) of LC/GC/MWCNT composite reduced by 15.9% and 10.7% compared with those of LC composite, respectively. This further confirmed that the additional MWCNT was beneficial to improving the fire retardancy of lignocellulose composites. The improved fire retardancy of LC/GC/MWCNT composite during combustion process may be that the addition of MWCNT promoted the formation of a char layer on the lignocellulose fibers [33,52].

As shown in Figure 9c,d, the total smoke production (TSP) and peak smoke production ratio (PSPR) of LC/GC/MWCNT composite reduced by 45.5% and 20.7% compared with those of LC composite, respectively, indicating that the additional MWCNT improved the smoke-suppression properties of lignocellulose composites. Some studies indicated that the density of MWCNT was about 2.2 g cm^−3^ and it had no change after combustion [53]. Therefore, the addition of MWCNT could decrease the emission of smoke composed of fine particles with low density during combustion process. Furthermore, some smoke gases may be adsorbed by MWCNT due to its high adsorption ability, which decreased the emission of smoke gases [54,55,56]. 

The fire retardant mechanism of MWCNT was further understood by evaluating the residue. As shown in Table 1, a higher char residue was observed in the LC/GC/MWCNT composite due to the addition of MWCNT, which agreed with the above TG results. The residue morphologies after combustion are shown in Figure 9e,f. The residue of LC composite only displayed a small amount of loose ash (Figure 9e). The residue of LC/GC/MWCNT composite exhibited several blocks with a compact structure (Figure 9f). Therefore, it is reasonable to deduce that the improvement in the fire retardancy and smoke suppression properties of LC/GC/MWCNT composite can be attributed to its compact structure and the formation of char layer during combustion process.

## 4. Conclusions

In summary, a novel composite based on lignocellulose/crosslinked chitosan/multiwalled carbon nanotube was prepared through hot-pressing process. The crosslinked chitosan and multiwalled carbon nanotube were used to enhance the mechanical properties, dimensional stabilities and fire retardancy of the lignocellulose composites. The results indicated that MOR, MOE, IB and TS of LC/GC/MWCNT composite could reach 35.3 MPa, 2789.1 MPa, 1.2 MPa and 22.4%, respectively. Additionally, the LC/GC/MWCNT composite exhibited improved thermal stability and fire retardancy. The total heat release and the total smoke production of LC/GC/MWCNT composite decreased by 10.7% and 45.5% compared with those of LC composite during cone calorimetry test. Such lignocellulose composite with improved mechanical strength, dimensional stability and fire retardancy is expected to be a promising candidate for green decorative materials.

## Figures and Tables

**Table 1:** Cone calorimeter data of LC composite and LC/GC/MWCNT composite.

Samples	PHRR ^a^	THR ^a^	TSP ^a^	PSPR ^a^	Residue ^a^
	(kW/m^2^)	(MJ/m^2^)	(m^2^/kg)	(m^2^/s)	(wt %)
LC	535.8 ± 8.6	27.0 ± 1.1	1.1 ± 0.1	0.029 ± 0.002	12.5 ± 1.3
LC/GC/MWCNT	450.9 ± 9.2	24.1 ± 0.9	0.6 ± 0.1	0.022 ± 0.002	19.7 ± 1.8

^a^ PHRR, THR, TSP, PSPR and Residue refer to peak heat release rate, total heat release, total smoke production, peak smoke production ratio and residue after cone tests, respectively.
